# Factors associated with chronic depressive symptoms across adolescence and young adulthood: a UK birth cohort study

**DOI:** 10.1017/S2045796024000350

**Published:** 2024-06-26

**Authors:** B. B. Durdurak, B. Williams, A. Zhigalov, A. Moore, P. Mallikarjun, D. Wong, S. Marwaha, I. Morales-Muñoz

**Affiliations:** 1 Institute for Mental Health, University of Birmingham, Edgbaston, Birmingham, UK; 2 School of Engineering and Technology, Aston University, Birmingham, UK; 3 Department of Psychiatry, University of Cambridge Herchel Smith Building for Brain & Mind Sciences, Cambridge, UK; 4 Early Intervention Service, Birmingham Women’s and Children’s NHS Trust, Birmingham, UK; 5 Leeds Institute of Health Sciences, School of Medicine, University of Leeds, Leeds, UK; 6 Specialist Mood Disorders Clinic, The Barberry Centre for Mental Health, Birmingham and Solihull NHS Trust, Birmingham, UK

**Keywords:** ALSPAC, depressive symptoms, factors, trajectories, young people

## Abstract

**Aims:**

Identifying children and/or adolescents who are at highest risk for developing chronic depression is of utmost importance, so that we can develop more effective and targeted interventions to attenuate the risk trajectory of depression. To address this, the objective of this study was to identify young people with persistent depressive symptoms across adolescence and young adulthood and examine the prospective associations between factors and persistent depressive symptoms in young people.

**Methods:**

We used data from 6711 participants in the Avon Longitudinal Study of Parents and Children. Depressive symptoms were assessed at 12.5, 13.5, 16, 17.5, 21 and 22 years with the Short Mood and Feelings Questionnaire, and we further examined the influence of multiple biological, psychological and social factors in explaining chronic depressive symptoms.

**Results:**

Using latent class growth analysis, we identified four trajectories of depressive symptoms: persistent high, persistent low, persistent moderate and increasing high. After applying several logistic regression models, we found that loneliness and feeling less connected at school were the most relevant factors for chronic course of depressive symptoms.

**Conclusions:**

Our findings contribute with the identification of those children who are at highest risk for developing chronic depressive symptoms.

## Introduction

Depression is one of the leading causes of mental health-related disability and is associated with social and economic burden (Patel *et al.*, 2016). Its onset usually occurs in mid-late adolescence (Solmi *et al.*, 2022). Depression in young people is an increasing concern not only because its onset occurs during a turbulent transitory period (Eyre *et al.*, 2021), but also because of its increasing prevalence, high recurrence rates and continuity into adulthood (Collishaw, 2015; Rice *et al.*, 2019; Thapar *et al.*, 2022). Depression can spontaneously remit, recur or persist especially among young people, due to substantial variability in the course of the illness in this group (Schubert *et al.*, 2017; Thapar *et al.*, 2022). Depression in this age group can also be the harbinger or first onset of other disorders such as bipolar disorder or schizophrenia (Ratheesh *et al.*, 2017; Thapar *et al.*, 2022). Although not all adolescents with significant psychopathology continue to have serious emotional problems in adulthood, many struggle with chronic and recurrent depression for extended periods of time, which is associated with poor adverse outcomes in the long term (Morales-Muñoz *et al.*, 2023). Many young people with severe and complex depression do not respond to first-line treatments and are at greater risk for suicidal ideation and attempts (Davey and McGorry, 2019). Therefore, characterising the differing trajectories of depressive symptoms over this critical period is crucial to aid assessment of prognosis and tailor early intervention strategies (Davey and McGorry, 2019; Marwaha *et al.*, 2021).

Although early intervention for depression in young people is still a blind spot (McGorry and Mei, 2018), a growing range of treatments and early intervention initiatives with evidence of efficacy for depression have been developed in the last decades (Hett *et al.*, 2021; Marwaha *et al.*, 2023). However, to enable potentially preventative approaches, further research is still needed to identify and target interventions towards the most salient factors associated with persistent depression to alter this worrying illness trajectory. To date, several candidate common clinical factors relevant to prevention and intervention for depression in young people have been detected, such as early and persistent adversity (between ages 9 and 11; Weavers *et al.*, 2021), parental psychopathology, irritability and anxiety (between ages 9 and 17; Rice *et al.*, 2017), gender, low socio-economic status (SES) and the quality of interpersonal relationships (between ages 4 and 17; Shore *et al.*, 2018), loneliness (between ages 9 and 18; Dunn and Sicouri, 2022), inflammation (until age 18; Toenders *et al.*, 2022a) and baseline severity of depressive symptoms and neuroticism (ages between 14 and 16; Toenders *et al.*, 2022b). However, there is still little consensus on what the most relevant modifiable factors (e.g., sleep-wake cycle patterns, early life stress) for depression in young people are and targeting modifiable factors is an additional, promising strategy for depression prevention (Marino *et al.*, 2021). It is crucial to identify early life factors in childhood that could be potentially modifiable, which would follow a recent study from our group that found that young people with persistent depression are at highest risk of adverse outcomes in young adulthood (Morales-Muñoz *et al.*, 2023).

Most studies in this area have focused on depression at a single time point, rather than exploring those individuals with chronic depression longitudinally. This approach does not capture intraindividual variability in symptoms or the longitudinal course of depressive symptoms (Kaup *et al.*, 2016). Various modifiable factors could contribute to an individual’s vulnerability to develop chronic depression, and understanding the composition of these modifiable factors is essential for planning effective prevention strategies (Avenevoli *et al.*, 2015). Therefore, identifying children and/or adolescents who are at highest risk for developing chronic depression and consequently further adverse outcomes is of utmost importance, so that we can develop more effective and targeted interventions to attenuate the risk trajectory of depression.

To address the current gap in the literature on depression in young people, the objectives of this study are to: (1) characterise the trajectories of depressive symptoms across adolescence and young adulthood from age 12.5 to 22 years; and (2) identify key potentially modifiable factors occurring in childhood before age 11 that associate with persistent high levels of depressive symptoms. We hypothesise that different trajectories of depressive symptoms are detectable across childhood and adolescence, including one with persistent depressive symptoms; and that several factors (e.g., poor sleep, feeling lonely or high levels of inflammation) in childhood would be associated with higher risk of persistent depressive symptoms across adolescence and young adulthood.

## Methods

### Participants

The Avon Longitudinal Study of Parents and Children (ALSPAC) is a UK birth cohort study, examining the determinants of development, health and disease during childhood and beyond (Boyd *et al.*, 2013; Fraser *et al.*, 2013; Northstone *et al.*, 2023). Pregnant women resident in Avon, UK with expected dates of delivery between 1 April 1991 and 31 December 1992 were invited to take part. The initial number of pregnant women enrolled was 14,541. Of these births, 13,988 children were alive at age 1 year. In addition, 913 children were enrolled after age 7 years, giving a total sample of 14,901 children. For this study, we used data from 6711 participants comprising offspring who had reported information on the depressive symptoms assessment at the age of 12.5 years old (see Figure S1 for a flow chart detailing sample definition). Further details of the ALSPAC are provided in Supplement. Ethical approval was obtained from the ALSPAC’s Law and Ethics Committee. Informed consent was obtained from the parents of the children.

### Measures

#### Depressive symptoms in young people

The Short Mood and Feelings Questionnaire (SMFQ; Angold *et al.*, 1995) was used to measure depressive symptoms at 12.5, 13.5, 16, 17.5, 21 and 22 years old. We selected these time points for two main reasons: (1) these were available within ALSPAC; (2) allows to cover key developmental stages from early adolescence, adolescence, late adolescence and young adulthood. SMFQ is a 13-item self-reported questionnaire enquiring about the occurrence of depressive symptoms over the past 2 weeks. The participants rate each statement as 2 (*true*), 1 (*sometimes true*) or 0 (*not true*), with total scores ranging from 0 (minimum) to 26 (maximum). Further, there is evidence supporting the validity of SMFQ to measure depression in young adults in the general population using ALSPAC (Eyre *et al.*, 2021), with Cronbach’s alpha = 0.92, and high accuracy for discriminating Major Depression Disordercases from non-cases (Area Under the Curve = 0.92). The commonly used cut-point in young people is ≥12 for screening for depression (Eyre *et al.*, 2021). Here, we used the SMFQ total score, with higher scores indicating greater depressive symptoms.

#### Factors

In this study, factors refer to several modifiable biological, psychological, environmental, social or family-related characteristics occurring in childhood before age 11 that precede and are associated with a higher likelihood of depression. We focus on factors occurring before age 11, as this is a critical transition period for most children in the UK for two main reasons: (1) this refers to the beginning of puberty on average (Roberts *et al.*, 2020) and (2) most children move from primary to secondary school in the UK. Accordingly, we selected the following factors available in ALSPAC for analysis, including *bullying, omega-3* and *parenting style* at 7 years, *diet* at 7.5 years, *intelligence quotient* (IQ) *and friendship quality* at 8 years, *childhood abuse* up to 8 years old, *locus of control* and *self-esteem* at 8.5 years, *engagement with arts, religious beliefs, inflammatory levels* (c-reactive protein and interleukin-6), *night-time sleep duration and bedtime* at 9 years, *loneliness, attentional switching, attentional control and selective attention* at 10 years and *participation in outdoor activities, school connectedness and school enjoyment* at 11 years. When selecting these factors, we used the existing list of factors suggested by Wellcome Trust (Abas, 2022; Wolpert *et al.*, 2021) and selected those available in ALSPAC in the first stage. We then finalised the list of relevant factors for this study following lived experience feedback.

A more detailed description of each of these factors appear in Table S1 in Supplement.

#### Confounders

Child’s sex, preterm delivery and temperament and parent-reported ethnicity (white and non-white) and SES were selected as covariates because of their impact on depression (Gelaye *et al.*, 2016; Harron *et al.*, 2021). Here, confounders occurred prenatally, at birth or during the first 2 years of life, and were non-modifiable or intrinsic variables (e.g., temperament, mental health), to differentiate from the factors, which occurred in childhood from 7 to 11 years old, and were all modifiable factors (i.e., more easily influenced from the outside). SES was mother-reported using the Cambridge Social Interaction and Stratification Scale (Stewart *et al.*, 1973). Prenatal maternal education was measured by asking mothers the highest qualification they achieved. Postnatal maternal depression (at 8 months) was measured using the Edinburgh Postnatal Depression Scale (Cox *et al.*, 1987). Finally, for child’s temperament at age 2, parents completed the Carey Infant Temperament Scale (Fullard *et al.*, 1984).

### Statistical analysis

A three-staged analysis plan was developed. In the first stage, descriptive analyses were conducted in SPSS, v29. Second, latent class growth analysis (LCGA) was conducted using Mplus-v8 (Muthén and Muthén, 2017) to assess trajectories of depressive symptoms across adolescence and young adulthood. The indicator variables were SMFQ total scores at 12.5, 13.5, 16, 17.5, 21 and 22 years. We fitted five models by increasing the number of classes (Jung and Wickrama, 2008; i.e., 2–6 classes). The best model was initially chosen based on fit indices (i.e., Bayesian information criteria [BIC] and Vuong–Lo–Mendell–Rubin [VLMR] test) as well as model entropy. Lower BIC values suggest better model fit and a significant VLMR value suggests that a (k)-class model fits the data better than a (k − 1)-class model. Entropy was used to select the best model fit in addition to BIC and VLMR; entropy with values approaching 1 indicates clear delineation of classes. We applied the full information maximum likelihood (FIML) method (Jung and Wickrama, 2008) which makes a missing at random assumption, permitting partially incomplete data to be included (Wardenaar, 2022). Third, we investigated the prospective associations between factors by age 11 and the trajectory of persistent high levels of depressive symptoms identified with LCGA (i.e., outcome), using logistic regression analyses. For the outcome, we created a dichotomous variable, based on the best model fit class obtained from LCGA: the class representing persistent high depressive symptoms was recoded as 1, while the other classes were recoded as 0. Additionally, we first tested unadjusted associations, and then we controlled for all the confounders in the adjusted model with each different factor. As primary analyses, we first tested these regression models with each factor as independent variable in separate models, and then we applied an additional regression analyses where we included a combination of these significant univariable factors together in the same model as factors, based on the feedback provided by young person with lived experience. More specifically, our co-author with lived experience in mental health (i.e., our lived experience lead) led several meetings with the Youth Advisory Group (YAG) from the University of Birmingham’s Institute for Mental Health, which comprises 18 young people aged 18–25 years old with mental health problems. During these meetings, our lived experience lead presented this study and the variables to be selected for the analyses to the YAG and received feedback regarding the most relevant factors for depression in young people based on lived experience. A list of factors was agreed at the end of these meetings between our lived experience lead and the YAG. These factors were highlighted as those that could have interactions with each other that made sense to individuals with lived experience of depression and were loneliness, IQ, school connectedness, school enjoyment, friendship, parenting and sleep. As secondary analyses, we conducted a logistic regression model in which we included as independent variables only those variables that were statistically significant in the separate regression analyses (from the primary analyses above). Further, we applied multinomial regression analyses, including as factors those from the combined logistic regression analyses (from the primary analyses above), and all the classes from the model with best model fit as the outcome. We used as reference the class with the largest sample size.

Finally, and as sensitivity analyses, we conducted the analyses above again excluding the loneliness item (‘I felt lonely’) from the SMFQ total score, to potentially control for any potential overlap between this item and our factor on loneliness.

As 57.1% of the original sample was lost to attrition at 12.5 years, we conducted logistic regressions to identify significant factors of attrition (see Supplementary, Table S2). Using the variables associated with selective dropout as the factors, we fitted a logistic regression model to determine weights for each individual using the inverse probability of response.

## Results

51% of our sample were female, 96.5% were White and 7.9% were born premature. Table 1 shows the frequencies and descriptive values of all the variables of interest in this study.

**Table 1.** Descriptive variables of our sample (*N* = 6711) (factors, outcomes and covariates)

	Frequencies		Descriptives		
**Socio-demographic factors**	*N*	%	Mean	SD	*N*
Male/female	3278/3418	49.0/51.0	—	—	—
White/non-white	5804/237	96.5/3.5	—	—	—
Length of pregnancy (weeks)	—	—	38.36	5.51	6368
Preterm delivery (yes/no)	317/3712	7.9/92.1	—	—	—
Maternal socio-economic status	—	—	55.08	13.40	5527
Temperament (mood score)	—	—	17.80	5.56	5744
**Sleep variables at 9 years**	*N*	%	Mean	SD	*N*
Bedtime weekend, hh:mm	—	—	21:30	0:40	5703
Bedtime weekday, hh:mm	—	—	20:50	0:36	5717
Total sleep weekday, h	—	—	10.45	0.65	5711
Total sleep weekend, h	—	—	10.43	0.82	5686
**Depressive symptoms variables**	*N*	%	Mean	SD	*N*
SMFQ, 12.5 years	—	—	3.97	3.86	6702
SMFQ, 13.5 years	—	—	4.88	4.49	5568
SMFQ, 16 years	—	—	5.83	5.58	3957
SMFQ, 17.5 years	—	—	6.48	5.21	3729
SMFQ, 21 years	—	—	5.56	5.45	2650
SMFQ, 22 years	—	—	6.07	5.42	2996
**Cognitive variables**	*N*	%	Mean	SD	*N*
WISC total IQ at 8 years	—	—	105.32	16.27	5722
Sky Search at 10 years	—	—	9.15	2.40	6170
Dual Task at 10 years	—	—	7.79	2.27	6065
Opposite Worlds (Same World) at 10 years	—	—	18.81	0.98	5914
Opposite Worlds (Opp. World) at 10 years	—	—	18.46	1.34	5912
**Inflammatory variables**	*N*	%	Mean	SD	*N*
Omega 3 fatty acids at 7 years	—	—	0.33	0.07	2333
CRP (mg/l) at 9 years	—	—	0.85	3.06	2185
IL-6 (pg/ml) at 9 years	—	—	3.25	0.71	1309
**Environmental and psychosocial** **variables**	*N*	%	Mean	SD	*N*
Bullying at 6.8 years, yes/no	603/2888	17.3/82.7	—	—	—
Female parenting score at 7 years	—	—	48.24	7.53	3614
Male parenting score at 7 years	—	—	33.30	10.79	3533
Friendship at 8 years	—	—	3.44	2.41	5580
Childhood abuse at 8 years, yes/no	501/4275	10.5/89.5	—	—	—
Self-esteem at 8.5 years	—	—	19.32	3.38	2945
Locus of control at 8.5 years			49.83	17.42	3055
Engagement with arts at 9 years, yes/no	4975/776	86.5/13.5	—	—	—
Religion beliefs at 9 years, yes/no	3591/2050	63.7/36.3	—	—	—
Loneliness at 10 years, yes/no	1971/4106	28.9/67.6	—	—	—
Participation outdoor act at 11 years, yes/no	1868/4225	30.7/69.3	—	—	—
School connectedness at 11 years	—	—	10.74	3.31	5315
School enjoyment at 11 years	—	—	6.14	1.84	5489

CRP = C-reactive protein, IL-6 = interleukin 6, SMFQ = Short Mood and Feelings Questionnaire, WISC = Wechsler Intelligence Scale for Children, IQ = intelligence quotient, *N* = number, SD = standard deviation.

### Latent classes of depressive symptoms

Table 2 shows VLMR, BIC and entropy for all five classes. Overall, a four-class model provided the best model fit. Although the five-class model had the lowest BIC and was statistically significant compared with the four-class model, the four-class model reported higher entropy value, suggesting a higher classification precision than class 5. Based on the class distinctiveness, clinical relevance and interpretability, the four-class model was identified as optimal for depressive symptoms. Additionally, the four-class model provided large enough (e.g., >3%) group sizes for each class.

**Table 2.** BIC, VLMR likelihood test *p*-values and entropy for Classes 2–6 of the SMFQ total score of depressive symptoms

	BIC	VLMR *p*-value	Entropy
2 classes	171,931.914	<0.001	0.810
3 classes	170,300.869	<0.001	0.776
4 classes	169,309.033	<0.001	0.762
5 classes	168,842.632	<0.001	0.759
6 classes	168,580.59	0.0561	0.715

BIC = Bayesian information criteria, VLMR = Vuong–Lo–Mendell–Rubin.

Number of cases LCGA – two classes: Class 1 = 1489 (17.1%), Class 2 = 7216 (82.9%).

Number of cases LCGA – three classes: Class 1 = 701 (8.1%), Class 2 = 1047 (12.0%), Class 3 = 6957 (79.9%).

Number of cases LCGA – four classes: Class 1 = 312 (3.6%), Class 2 = 1010 (11.6%), Class 3 = 910 (10.5%), Class 4 = 6473 (74.4%).

Number of cases LCGA – five classes: Class 1 = 211 (2.4%), Class 2 = 258 (3.0%), Class 3 = 977 (11.2%), Class 4 = 1046 (12.0%), Class 5 = 6213 (71.4%).

Number of cases LCGA – six classes: due to VLMR *p*-value > 0.050, a model with six classes was not detected.

**Figure 1.**
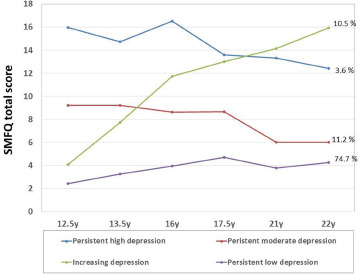
Growth trajectories of depressive symptoms across childhood to adolescence. The latent class growth analyses detected a best model fit for four classes. Class 1 (blue line on the top) represents individuals with persistent high levels of depressive symptoms across time points. Class 2 (red line in the middle) represents individuals with persistent moderate levels of depressive symptoms. Class 3 (green line) represents individuals with increasing levels of depressive symptoms. Class 4 (purple line on the bottom) represents individuals with persistent low levels of depressive symptoms.

Figure 1 shows the trajectories of the four-class model. Class 1 ‘persistent high levels of depressive symptoms’ (3.6%) was characterised by a chronic course of depressive symptoms, with the highest burden of depressive symptoms. Class 2 ‘moderate levels of depressive symptoms’ (11.2%) described a reducing moderate trajectory. Class 3 ‘increasing levels of depressive symptoms’ (10.5%) showed a gradual increase of symptoms. Finally, Class 4 ‘persistent low levels of depressive symptoms’ (74.7%) had a persistent lower level of course trajectory.

### *Factors* *and persistent high levels of depressive symptoms*

When we applied separate logistic regression models for each factor, we found that several factors were significantly associated with higher levels of depressive symptoms in the adjusted model (see Table 3). More specifically, higher loneliness score at 10 (OR, 2.01; 95% CI, 1.51–2.67; *p* < 0.001); lower participation in outdoor activities at 11 (OR, 0.57; 95% CI, 0.38–0.86; *p* = 0.007); lower attentional control at 10 (OR, 0.91; 95% CI, 0.85–0.97; *p* = 0.006); lower IQ at 8 (OR, 0.98; 95% CI, 0.97–0.99; *p* < 0.001); feeling less connected with the school at 11 (OR, 1.19; 95% CI, 1.15–1.23; *p* < 0.001); lower school enjoyment at 11 (OR, 1.26; 95% CI, 1.17–1.35; *p* < 0.001); lower friendship quality at 8 (OR, 1.12; 95% CI, 1.06–1.18; *p* < 0.001); health conscious/vegetarian diet at 8.6 (OR, 1.22; 95% CI, 1.05–1.41; *p* = 0.009) and worse paternal parenting at 7 (OR, 1.02; 95% CI, 1.00–1.04; *p* = 0.032) were all significantly associated with persistent high levels of depressive symptoms across adolescence and young adulthood.

**Table 3.** Associations between factors and persistent high levels of depressive symptoms from 12.5 months to 22 years, in separate models per active risk factor

	Persistent high levels of depressive symptoms					
	Unadjusted model			Adjusted model*		
	OR	95% CI	*p*	OR	95% CI	*p*
Female parenting score at 81 months**	1.02	0.99, 1.04	0.164	1.02	1.00, 1.05	0.111
Male parenting score at 81 months**	**1.02**	**1.00, 1.03**	**0.048**	**1.02**	**1.00, 1.04**	**0.032**
Bullying at 6.8 years	1.04	0.70, 1.54	0.844	1.13	0.76, 1.69	0.542
Omega-3 at 7.5 years	5.16	0.23, 114.7	0.300	4.61	0.15, 144.21	0.385
Childhood abuse at 8 years	0.92	0.56, 1.49	0.725	0.94	0.56, 1.57	0.813
Friendship at 8 years**	**1.11**	**1.06, 1.17**	**<0.001**	**1.12**	**1.06, 1.18**	**<0.001**
Self-esteem at 8 years	1.05	0.99, 1.12	0.087	1.05	0.99, 1.12	0.121
Locus of control at 8 years	1.01	1.00, 1.02	0.345	1.00	0.99, 1.01	0.512
WISC total IQ at 8 years	**0.98**	**0.98, 0.97**	**<0.001**	**0.98**	**0.97, 0.99**	**<0.001**
Art engagement at 9 years	1.38	0.78, 2.43	0.264	1.39	0.79, 2.48	0.265
C-reactive protein at 9 years	0.99	0.92, 1.06	0.746	0.98	0.90, 1.06	0.587
Interleukin-6 at 9 years	0.91	0.62, 1.34	0.624	0.91	0.60, 1.40	0.677
Total sleep during night at 9 years	0.85	0.55, 1.30	0.440	0.87	0.69, 1.08	0.212
Bedtime at 9 years	1.11	0.70, 1.76	0.670	1.10	0.87, 1.38	0.420
Religion at 9 years	**1.44**	**1.05, 1.96**	**0.022**	1.15	0.83, 1.58	0.397
Attention Sky Search at 10 years	1.00	0.94, 1.07	0.972	0.98	0.92, 1.02	0.155
Attention Dual Task at 10 years	1.00	0.94, 1.07	0.943	0.97	0.93, 1.01	0.129
Attention Opposite Worlds Task at 10 years	**0.88**	**0.80, 0.97**	**0.009**	**0.91**	**0.85, 0.97**	**0.006**
Loneliness at 10 years	**2.04**	**1.56, 2.67**	**<0.001**	**2.01**	**1.51, 2.67**	**<0.001**
School connectedness at 11 years**	**1.17**	**1.13, 1.21**	**<0.001**	**1.19**	**1.15, 1.23**	**<0.001**
School enjoyment at 11 years**	**1.17**	**1.09, 1.26**	**<0.001**	**1.26**	**1.17, 1.35**	**<0.001**
Participation outdoor activities at 11 years	**0.56**	**0.38, 0.84**	**0.005**	**0.57**	**0.38, 0.86**	**0.007**

OR = odds ratio.

* Adjusted model controlled for sex, ethnicity, SES, temperament at 2 years and preterm, and maternal postnatal depression at 8 months.

** These variables were invertedly coded, with higher scores indicating worse outcomes, and lower scores better outcomes.

Each predictor was included in separate regression analyses together with the covariates (for the adjusted models).

### Associations between combination of factors with persistent high levels of depressive symptoms

We also tested the associations between a combination of factors (which was created based on the feedback by lived experience, rather than using other statistical approaches such as factor analyses) with persistent high levels of depressive symptoms across adolescence and young adulthood (see Table 4). In the adjusted model, we found that only higher loneliness (OR, 2.02; 95% CI, 1.11–3.67; *p* = 0.022) and lower school connectedness (OR, 1.29; 95% CI, 1.16–1.43; *p* < 0.001) were significantly associated with persistent high levels of depressive symptoms across adolescence and young adulthood.

**Table 4.** Associations between combined factors and persistent high levels of depressive symptoms

	Persistent high levels of depressive symptoms					
	Unadjusted model			Adjusted model*		
	OR	95% CI	*p*	OR	95% CI	*p*
Male parenting score at 81 months**	1.00	0.97−1.03	0.825	1.00	0.97−1.03	0.924
Friendship at 8 years**	0.98	0.87−1.11	0.763	0.96	0.84−1.08	0.478
WISC—total IQ at 8 years	0.99	0.97−1.01	0.240	0.99	0.97−1.02	0.625
Total sleep during night at 9 years	1.62	0.60−4.33	0.340	1.68	0.59−4.74	0.329
Bedtime at 9 years	1.81	0.62−5.33	0.281	1.60	0.51−5.03	0.418
**Loneliness at 10 years**	**2.25**	**1.30−3.89**	**0.004**	**2.02**	**1.11−3.67**	**0.022**
School connectedness at 11 years**	**1.24**	**1.13−1.36**	**<0.001**	**1.29**	**1.16−1.43**	**<0.001**
School enjoyment at 11 years**	**0.75**	**0.60−0.94**	**0.011**	0.81	0.62−1.03	0.112

OR = odds ratio.

* Adjusted model controlled for sex, ethnicity, SES, temperament at 2 years, preterm and maternal postnatal depression at 8 months.

** These variables were invertedly coded, with higher scores indicating worse outcomes, and lower scores better outcomes. Note 1: The selection of these factors was done based on lived experience involvement. Here, all the factors were included together within the same regression analyses model.

As a sensitivity analyses, we conducted an additional regression analyses model, where we included in the same model all the factors that appeared statistically significant in the separate regression analyses models (from Table 3). Importantly, we again found that only higher loneliness (OR, 2.20; 95% CI, 1.18–4.11; *p* = 0.013) and lower school connectedness (OR, 1.33; 95% CI, 1.19–1.49; *p* < 0.001) were significantly associated with persistent high levels of depressive symptoms, which supports the robustness of our results (see Table S3, Supplement).

The results from the multinomial regression analyses when we compared class 1 (i.e., our class of interest) versus class 4 (i.e., reference class) showed similar results as above, with higher loneliness (OR, 1.71; 95% CI, 1.24–2.36; *p* < 0.001) and lower school connectedness (OR, 1.02; 95% CI, 1.00–1.03; *p* = 0.004) being the only significant factors (see Table S4, Supplement).

Finally, our sensitivity analyses when we excluded the item on loneliness from the SMFQ total score reported similar results. Briefly, similar trajectories of depressive symptoms were reported, with a four-classes model providing the best model fit. Further, loneliness and lack of connection with school were still the only factors that were significantly associated with persistent high levels of depressive symptoms when we combined relevant factors together in the analyses. Further details are provided in Supplement (Tables S5–S8 and Figure S2).

## Discussion

**Figure 2.**
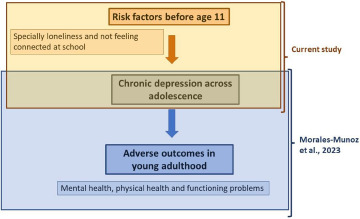
Model of depressive symptoms across adolescence, risk factors and impacts. Here we present how specific risk factors before age 11 (and especially loneliness and not feeling connected at school) lead to chronic depressive symptoms across adolescence, which subsequently leads to the development of a range of adverse outcomes in young adulthood, including mental health, physical health and functioning problems. On top (in brown colour) we present the main purpose of this current study, while on the bottom (in blue colour) we present the main findings of our recent study (Morales-Muñoz *et al.*, 2023). More specifically, in our recent study, we found that chronic depression across adolescence led to a range of mental health (psychotic disorder, severe depression, generalised anxiety disorder and panic disorder), physical health (asthma, arthritis and heart problems) and functioning problems (not being in education/employed/training), all at 24 years old.

Using data from a large population-based cohort study, we identified four different trajectories of depressive symptoms across adolescence and young adulthood. Further, we detected a range of modifiable factors in childhood that were associated with increased risk of developing high levels of depressive symptoms across adolescence and young adulthood. Both findings were consistent with our initial hypotheses. We build on our previous work on the long-term adverse outcomes associated with chronic depressive symptoms and examined what factors might explain this (see Fig. 2).

First, we found four different trajectories of depressive symptoms across adolescence and young adulthood including persistent high and persistent low levels, which is consistent with previous work (Vannucci and McCauley Ohannessian, 2018; Weavers *et al.*, 2021). More specifically, similar to our findings, all of the previous studies above detected a low stable group which represented the vast majority of the sample. Persistently high depression group trajectories have also been found in previous research supporting the relevance of chronicity of depression in youth (Bulhões *et al.*, 2021; Kwong *et al.*, 2019; Shore *et al.*, 2018; Weavers *et al.*, 2021). Further, in line with previous research (Kwong *et al.*, 2019; Weavers *et al.*, 2021), increasing levels of depressive symptoms and persistent levels of depressive symptoms differed in their age of onset, with an earlier age (starting at or before age 12.5 years) for persistent levels than increasing levels of depressive symptoms (starting at age 16 years). However, some other studies have reported slightly different depression trajectories to ours. For example, Cumsille *et al.* (2015), Duchesne and Ratelle (2014), Essau *et al.* (2020), Vannucci and McCauley Ohannessian (2018) and Weavers *et al.* (2021) detected remitting trajectories. It is likely that the differences may arise due to methodological variations across the studies, such as age range (e.g., Essau *et al.*, 2020), measures used (e.g., Vannucci and McCauley Ohannessian, 2018), number of assessments (e.g., Bulhões *et al.*, 2021), duration of the follow-up (e.g., Duchesne and Ratelle (2014) and confounders included (e.g., Ferro *et al.*, 2015).

Second, in relation to the associations between factors in childhood and persistent high levels of depressive symptoms across adolescence and young adulthood, we found that overall, the most relevant factors were loneliness and not feeling connected with the school, which is consistent with previous research in the field. For example, previous research found that family and school connectedness were negatively associated with depression and suicidal ideation (Arango *et al.*, 2019) and that increasing school connectedness should be considered as a universal adolescent mental health strategy (Allen *et al.*, 2022; Langille *et al.*, 2015). Further, the existing evidence supports that higher levels of loneliness are linked to higher levels of depressive symptoms in children and adolescents (Dunn and Sicouri, 2022), and that childhood loneliness is a major predictor for anxiety and depressive disorders in young adults (Xerxa *et al.*, 2023), providing some external validation of our results. Some of the potential explanations for why loneliness and social connectedness were the most relevant factors for chronic depression in our study might be found in the fact that adolescence is a period in which the social brain undergoes structural development, such as heightened self-awareness and social understanding (Kilford *et al*., 2016). Adolescents, while developing cognitive maturation, start to form more complex and hierarchical peer relationships and are more sensitive to acceptance and rejection by their peers compared to children (Kilford *et al*., 2016; King *et al.*, 2018). Perceived social connectedness and loneliness may thus be key. It is suggested that the problem of current prevention of depression is that it is not structurally and socially embedded (Ormel *et al.*, 2019). Schools and colleges may be very suitable settings for identifying and treating young people at highest risk of developing chronic depression, although continued optimisation and refinement of school based interventions is needed to enhance their impact (Werner-Seidler *et al.*, 2021). However, the bidirectional associations of these two factors with depression should be taken into consideration when interpreting our results, as both loneliness (Achterbergh *et al.*, 2020) and lack of connection with the school (Marraccini and Brier, 2017) are also considered a symptom for depression in young people. In the current study, we explored and found evidence that both factors precede the development of chronic depressive symptoms from childhood to adulthood, but future studies should further explore the prospective associations of chronic depression in young people with loneliness and school connectedness.

### Implications for practice

There are several implications. Firstly, the timing of the onset of depression is important in chronic course; the earlier depression starts, the greater the risk that it could be chronic (Thapar and Riglin, 2020). Chronicity is not only important because of individual and family suffering, and social consequences, but because chronic depressive trajectories in youth are associated with transition to other severe mental disorders (Hartmann *et al.*, 2019; McGorry *et al.*, 2018; Ratheesh *et al.*, 2023) such as bipolar disorder (Durdurak *et al.*, 2022; Ratheesh *et al.*, 2017). The same trajectory patterns for increasing and persistent classes have also been detected in older adult communities (Mirza *et al.*, 2016), suggesting patterns of chronicity are similar across the lifespan. Secondly, the identified factors have direct clinical and childhood policy relevance. Screening for clinically relevant depressive symptoms among children and providing early intervention at schools may be an effective strategy to reduce the burden of disease from depression in children and adolescents (Caldwell *et al.*, 2019; Garcia-Carrion *et al.*, 2019) while also looking at the role of school personnel in the detection, referral and provision of help for youth psychopathology due to their influence on the outcomes (Werner-Seidler *et al*., 2021). Thirdly, the timing of such interventions should be a central component of these efforts, and our findings suggest that these early interventions should start as early as 11-year-old, which is a key transition period for many children. Since universal interventions are less effective than targeted intervention (Werner-Seidler *et al*., 2021) and depression in children and adolescents is considerably undertreated (Mojtabai *et al.*, 2016), the development of youth-specific specialist integrated mental health services for young people is particularly crucial for the public mental health service systems which would strengthen existing child and adolescent services (Mcgorry *et al.*, 2007). Our findings support the widespread call for an investment in young people’s mental health, by identifying where and when this might be targeted to prevent chronicity of depressive symptom burden (Kieling *et al.*, 2024).

### Strengths and limitations

Strengths of this study include the repeated assessment of depressive symptoms from adolescence to young adulthood and the broad assessment of factors for depressive symptoms in a large population-based sample. Further, a young person with lived experience from our team together with a wider group of young people with lived experience provided substantial feedback and insight including definition of the research priorities, selection of factors and interpretation of our findings. However, our study has also some limitations. First, the majority of participants were of white ethnicity, which limits the generalisability of our findings to other ethnic groups. Second, although we controlled for maternal postnatal depression in this study, we did not look at the role of anxiety and other parental psychopathology which are factors of major depressive disorders (stages 0–2; Hartmann *et al.*, 2019). Given that this is a birth cohort study and not a high-risk study, the role of parental psychopathology is something that needs further exploration in future studies, especially in high-risk population, rather than the general population. Third, since the informant differed for the assessment of some of the factors (i.e., parent-reported vs self-reported), clinically relevant symptoms might have been missed such as childhood trauma and bullying, which were parent-reported. Further, depression was assessed using a self-reported questionnaire (SMFQ) rather than a direct interview. Although SMFQ is considered a valid instrument to measure depression in young people (Eyre *et al.*, 2021), this is still subject to potential bias. Fourth, although this work was co-produced with a young person with lived experience liaising with a small group of young people with lived experience from Birmingham area, this could be also subject to some bias as it was limited to a specific group of people. Fifth, although a relatively large number of factors were examined, some other relevant factors (e.g., anxiety, neurodevelopmental conditions, genetic factors, family history of psychopathology) were not included (Maciejewski *et al.*, 2018; Rice *et al*., 2017; Vidal-Ribas *et al.*, 2016). In addition, other relevant confounders (such as sexual orientation) were not available within our dataset, and thus we were not able to control for them. Sixth, LCGA do not necessarily identify and reflect the true clinical sub-populations, but rather those that fit optimally according to currently default criteria for evaluation model fit in Mplus (Arnold *et al.*, 2014). Seventh, since there were high rates of attrition, this might have caused bias in estimates of the associations we found (Cornish *et al.*, 2021). However, to be able to reduce this bias we have utilised FIML and inverse probability weighting methods. Another bias that might have arisen in our findings is the previously detected lack of measurement invariance for the SMFQ assessment at age 12.5 in ALSPAC (Schlechter *et al.*, 2023). Eighth, although working with people with lived experience in mental health research carries a wide range of benefits, lived experience perspectives could be criticised as being limited by their subjectivity (Davis *et al.*, 2024). However, majority of the research relies on background assumptions and involving lived experience work in research can increase the relevance, feasibility, adoption, implementation and sustainability of research, particularly in mental health research (Davis *et al.*, 2024).

## Conclusion

Our findings support the existence of different trajectories of depressive symptoms across adolescence and young adulthood, including a group of young people with persistent high depressive levels. Further, we found that loneliness and social connection before age 11 were the most relevant factors for chronic depressive symptoms in young people and these could be addressed in depression prevention programs. Our findings contribute to the existing research in depression with the identification of those children who are at highest risk for developing persistent depressive symptoms. Prevention has been the most neglected aspect of depression, and our findings add to growing evidence about the urgent need of improving early intervention strategies to prevent the experience of chronic depression in adulthood.

## Supporting information

Durdurak et al. supplementary material Durdurak et al. supplementary material

## Data Availability

Access to ALSPAC data is through a system of managed open access (http://www.bristol.ac.uk/alspac/researchers/access/).

## Supplementary material

The supplementary material for this article can be found at https://doi.org/10.1017/S2045796024000350.
