# Supplementary material for: Factors associated with chronic depressive symptoms across adolescence and young adulthood: a UK birth cohort study
Source: Epidemiol Psychiatr Sci. 2024 Jun 26;33:e32. doi: 10.1017/S2045796024000350 (PMC11362687; doi:10.1017/S2045796024000350)
Supplement: Durdurak et al. supplementary material [file S2045796024000350sup001.docx]

**Figure S1.** Flow chart of participation in the Avon Longitudinal Study of Parents

and Children. ALSPAC: Avon Longitudinal Study of Parents and Children; SMFQ:

The Short Moods and Feelings Questionnaire

Pregnancies enrolled in ALSPAC Phase 1

**N = 14,541**

Exclusions (n =922)

Multiple births

Not alive at 1 year

Base sample after exclusions

**n= 13,988**

An additional 913 children enrolled

Sample after bolstering the initial sample **(n=14901)**

Child invited to complete SMFQ when 12.5 years old **(n=6711)**

**Table S1.** Factors, time points when they were assessed, and the measurements used.

| **Factors** | **Time Points** | **Measurement** |
| --- | --- | --- |
| Bullying | 6.8 years | Parent-reported items on whether the child had been bullied in the last 6 months when child was at 6-7 years old.  A dichotomous score was used (1=Yes/ 0=No). Bullying is considered a risk factor. |
| Parenting Skills | 7 years | Parent-reported - Mothers reported their attitudes, behaviours  and feelings toward the study child. Higher scores indicate least good/positive parenting skills (i.e., risk factor), while lower scores indicate best parenting skills (i.e., protective factor). |
| Omega-3 | 7.5 years | Blood sample collected from non-fasting participants. Overall, higher levels of Omega-3 are considered a protective factor, while lower levels a risk factor. |
| Childhood Abuse | 8 years | Parent-reported items on whether the child was sexually abused and/or physically abused from birth up to 8 years old.  A dichotomous score was used (1=Yes/ 0=No). Childhood abuse is considered a risk factor. |
| Friendship | 8 years | Self-reported measure, using the Cambridge Hormones and Moods Project Friendship questionnaire (Goodyer, Wright, &  Altham, 1989). Higher scores indicate worse friendship quality (i.e., risk factor), while lower scores indicate better friendship quality (i.e., protective factor). |
| Intelligence Quotient (IQ) | 8 years | The Wechsler Intelligence Scale for Children (WISC-III; Wechsler, 1949). Higher scores indicate higher IQ levels (i.e., protective factor), while lower score indicate lower IQ levels (i.e., risk factor). |
| Locus of Control | 8.5 years | Self-reported - The Nowicki-Strickland Internal-External  scale (NSIE; Nowicki & Strickland, 1973). Higher scores reflect a more external locus of control (risk factor), while lower scores indicate more internal locus of control (protective factor). |
| Self-esteem | 8.5 years | Self-reported - The sixth subscale of Harter’s Self Perception Profile for Children (Harter, 1985). Higher scores indicate higher self-esteem (i.e., protective factor), while lower scores indicate lower self-esteem (i.e., risk factor). |
| C-Reactive Protein (CRP) | 9 years | Automated particle-enhanced immunoturbidimetric assay (Khandaker et al., 2014). Elevated levels of CRP are considered risk factors, while lower levels protective factors. |
| Engagement with Arts | 9 years | Parent-reported item “Child likes playing instrument, singing, dancing, reading and/or drawing”. A dichotomous score was  used (Yes/No). Engagement with arts is considered a protective factor. |
| Interleukin-6 (IL-6) | 9 years | Enzyme-linked immunosorbent assay. Elevated levels of IL-6 are considered risk factors, while lower levels protective factors. |
| Night-time Sleep Duration and Bedtime | 9 years | Parent-reported item “Time child usually goes to sleep”; “Time child usually gets up”: Night-time sleep duration was extracted from these two measures. Shorter sleep duration is considered a risk factor while longer sleep duration a protective factor. |
| Religious Beliefs | 9 years | Parent-reported item “Child takes an interest in religion”. Parents could answer to this by choosing 4 options; 1 = Yes, very interested, 2 = Yes, somewhat interested, 3 = No, not interested, 4 = Not sure. We then created a dichotomous variable where 1=Yes, very interesting; and Yes, somewhat interested, and 0=No, not interested. Religious belief is overall considered a risk factor. |
| Attentional Switching | 10 years | The dual-attention task of ‘Sky Search’ subtest of the adapted Test-of-Everyday-Attention-for-Children (TEA-Ch; Robertson et al., 1996). Higher scores indicate greater attentional switching (protective factor), while lower scores indicate poorer attentional switching (risk factor). |
| Attentional Control | 10 years | The ‘Opposite Worlds’ task from the TEA-Ch (Robertson et  al., 1996). Higher scores indicate greater attentional control (protective factor), while lower scores indicate poorer attentional control (risk factor). |
| Loneliness | 10 years | Self-reported question - The item was part of the SMFQ, asking children whether they felt lonely in the last two weeks. Scoring: 1 = True, 2 = Sometimes, 3 = Not at all. We then created a dichotomized score where 1=True and sometimes,  and 0=Not at all. Loneliness is considered a risk factor. |
| Selective Attention | 10 years | The ‘Sky Search’ Task (TEA-Ch; Robertson et al., 1996).  Higher scores indicate better selective attention (protective factor), while lower scores indicate poorer selective attention (risk factor). |
| School connectedness | 11 years | Self-reported questions about the child’s sense of belonging at the school and how positively they believe they are viewed by others. Higher scores indicate less school connection (risk factor), while lower scores indicate more school connection (protective factor). |
| School enjoyment | 11 years | Self-reported questions about the child’s enjoyment of classes  and school. Higher scores indicate less school enjoyment (risk factor), while lower scores indicate more school enjoyment (protective factor). |
| Participation in outdoor activities | 11 years | Parent-reported item “Child does other after-school activities with friends”. A dichotomous score was used (Yes/No). Outdoor activities is considered a protective factor. |

**Table S2.** Differences in socio-demographic variables between non-participating and participating subjects in the study at 10 years old

# Non-participating group in the study Participating group in the study Participating versus

**Non-participating**

|  | *Mean* | *SD* | *Mean* | *SD* | *OR (95% CI)* | *p* |
| --- | --- | --- | --- | --- | --- | --- |
| Maternal age when born | 26.81 | 5.11 | 29.05 | 4.58 | 1.10 (1.09 to 1.11) | <0.001 |
| Gestational age | 37.24 | 7.46 | 39.45 | 1.85 | 1.14 (1.10 to 1.13) | <0.001 |
| Birth weight, kg | 3.34 | 0.62 | 3.42 | 0.54 | 1.28 (1.21 to 1.35) | <0.001 |
| Family Adversity score | 5.34 | 4.75 | 3.93 | 4.01 | 0.93 (0.92 to 0.94) | <0.001 |
| **Non-participating group in the study Participating group in the study** | | | | | | |
|  | *N* | *%* | *N* | *%* |  |  |
| Sex |  |  |  |  | 0.94 (0.88 to 1.01) | 0.077 |
| Male / Female | 3772 / 3498 | 51.9 / 48.1 | 3919 / 3850 | 50.4 / 49.6 |  |  |
| Ethnicity |  |  |  |  | 0.42 (0.33 to 0.52) | <0.001 |
| White / Other | 4985 / 205 | 96.1 / 3.9 | 7077 / 122 | 98.3 / 1.7 |  |  |
| Preterm delivery |  |  |  |  | 0.56 (0.49 to 0.65) | <0.001 |
| Yes / No | 509 / 3443 | 12.9 / 87.1 | 347 / 4169 | 7.7 / 92.3 |  |  |

The individuals associated with attrition at 12.5 years were more frequently born preterm, had lower weight at birth, had shorter gestational age, had younger mothers at birth, had higher scores in family adversity, and were more often of non-white ethnicity.

**Table S3.** Associations between statistically significant risk factors and persistent high levels of depressive symptoms from 12.5 months to 22 years.

| **Persistent high levels of depressive symptoms** | | | | | | |
| --- | --- | --- | --- | --- | --- | --- |
|  | Unadjusted model | | |  | Adjusted model | |
|  | OR | 95% CI | p | OR | 95% CI | p |
| Male Parenting Score at 81 months | 0.99 | 0.96 to 1.04 | 0.891 | 1.00 | 0.97 to 1.04 | 0.778 |
| Friendship at 8 years | 0.94 | 0.79 to 1.12 | 0.473 | 0.92 | 0.80 to 1.05 | 0.213 |
| WISC – Total IQ at 8 years | 0.98 | 0.95 to 1.01 | 0.286 | 0.99 | 0.97 to 1.01 | 0.420 |
| Attention Opposite Worlds Task at 10 years | 1.30 | 0.53 to 3.20 | 0.561 | 1.15 | 0.57 to 2.29 | 0.695 |
| **Loneliness at 10 years** | **2.32** | **1.09 to 4.97** | **0.030** | **2.20** | **1.18 to 4.11** | **0.013** |
| **School Connectedness at 11 years** | **1.27** | **1.12 to 1.45** | **<0.001** | **1.33** | **1.19 to 1.49** | **<0.001** |
| School Enjoyment at 11 years | 0.73 | 0.53 to 1.01 | 0.055 | 0.76 | 0.54 to 1.07 | 0.121 |
| Participation outdoor activities at 11 years | 0.66 | 0.22 to 2.01 | 0.468 | 0.77 | 0.33 to 1.79 | 0.549 |

**Note1: Here we included all the factors that appeared statistically significant in the regression analyses conducted with each predictor in separate model (i.e., Table 3).

**Table S4.** Multinomial adjusted regression analyses between factors and each of the LCGA classes

|  | **Persistent night-time short sleep** | | |
| --- | --- | --- | --- |
| **Factors** | *OR* | *CI 95 %* | *p* |
|  | **Class 1 Vs Class 4** | | |
| Male Parenting Score at 81 months | 1.01 | 0.99 – 1.03 | 0.068 |
| Friendship at 8 years | 1.02 | 0.95 – 1.10 | 0.536 |
| WISC – Total IQ at 8 years | 1.05 | 0.99 – 1.12 | 0.127 |
| Total Sleep During Night at 9 years | 1.12 | 0.67 – 1.88 | 0.654 |
| Bedtime at 9 years | 0.87 | 0.48 – 1.58 | 0.644 |
| **Loneliness at 10 years** | **1.71** | **1.24 – 2.36** | **<0.001** |
| **School Connectedness at 11 years** | **1.02** | **1.00 – 1.03** | **0.004** |
| School Enjoyment at 11 years | 1.06 | 0.94 – 1.19 | 0.322 |
|  | **Class 2 Vs Class 4** | | |
| **Male Parenting Score at 81 months** | **1.02** | **1.00 – 1.03** | **0.019** |
| Friendship at 8 years | 0.96 | 0.90 – 1.03 | 0.298 |
| WISC – Total IQ at 8 years | 0.99 | 0.99 – 1.01 | 0.549 |
| Total Sleep During Night at 9 years | 1.01 | 0.63 – 1.61 | 0.978 |
| Bedtime at 9 years | 0.99 | 0.58 – 1.69 | 0.978 |
| **Loneliness at 10 years** | **2.77** | **2.01 – 3.66** | **<0.001** |
| **School Connectedness at 11 years** | **1.06** | **1.01 – 1.12** | **0.024** |
| School Enjoyment at 11 years | 1.10 | 0.99 – 1.22 | 0.086 |
|  | **Class 3 Vs Class 4** | | |
| Male Parenting Score at 81 months | 1.01 | 0.98 – 1.04 | 0.618 |
| Friendship at 8 years | 0.96 | 0.84 – 1.09 | 0.516 |
| WISC – Total IQ at 8 years | 0.99 | 0.98 – 1.02 | 0.795 |
| Total Sleep During Night at 9 years | 1.73 | 0.61 – 4.95 | 0.305 |
| Bedtime at 9 years | 1.57 | 0.49 – 5.00 | 0.443 |
| **Loneliness at 10 years** | **2.83** | **1.54 – 5.21** | **<0.001** |
| **School Connectedness at 11 years** | **1.32** | **1.19 – 1.46** | **<0.001** |
| School Enjoyment at 11 years | 0.81 | 0.64 – 1.02 | 0.073 |

Class 4 (i.e., persistent low levels of depression) was treated here as the reference class, as this is the class with higher number of cases.

Class 1 “persistent high levels of depressive symptoms”; Class 2 “moderate levels of depressive symptoms”; Class 3 “increasing levels of depressive symptoms”; Class 4 “persistent low levels of depressive symptoms” (41.4%).

|  | **BIC** | **VLMR p-value** | **Entropy** |
| --- | --- | --- | --- |
| 2 classes | 153116.541 | <0.001 | 0.843 |
| 3 classes | 152314.549 | <0.001 | 0.813 |
| 4 classes | 151341.679 | <0.001 | 0.859 |
| 5 classes | 152368.808 | 0.4978 | 0.574 |
| 6 classes | 151829.476 | 0.4898 | 0.747 |

**Table S5.** BIC, VLMR Likelihood Test p Values, and Entropy for Classes 2–6 of the SMFQ total score of Depressive symptoms, without loneliness item.

BIC=Bayesian information criteria; VLMR=Vuong Lo-Mendel-Rubin


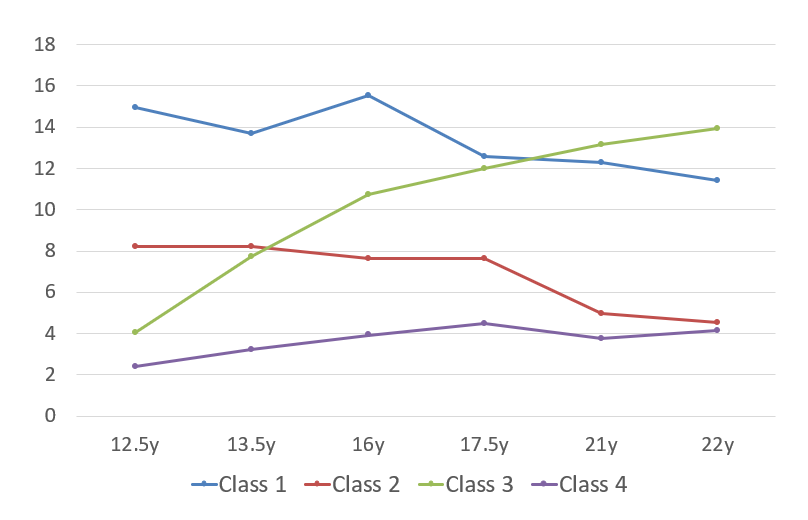


**Figure S1.** **Growth trajectories of depressive symptoms across childhood to adolescence, without loneliness item.** The latent class growth analyses detected a best model fit for 4 classes. Class 1 (blue line on the top) represents individuals with persistent high levels of depressive symptoms across time points. Class 2 (red line in the middle) represents individuals with persistent moderate levels of depressive symptoms. Class 3 (green line) represents individuals with increasing levels of depressive symptoms. Class 4 (purple line on the bottom) represents individuals with persistent low levels of depressive symptoms.

**Table S6.** Associations between factors and persistent high levels of depressive symptoms from 12.5 months to 22 years, in separate models per active risk factor, without loneliness items, without loneliness item.

|  | **Persistent high levels of depressive symptoms** | | | | | |
| --- | --- | --- | --- | --- | --- | --- |
|  | Unadjusted model | | |  | Adjusted model | * |
|  | OR | 95% CI | p | OR | 95% CI | p |
| Female Parenting Score at 81 months** | 1.02 | 0.99 – 1.04 | 0.210 | 1.02 | 0.99 – 1.05 | 0.111 |
| Male Parenting Score at 81 months** | **1.02** | **1.00 – 1.03** | **0.045** | **1.02** | **1.00 – 1.04** | **0.032** |
| Bullying at 6.8 years | 1.11 | 0.75 – 1.65 | 0.587 | 1.13 | 0.76 – 1.69 | 0.542 |
| Omega-3 at 7.5 years | 6.04 | 0.22 –62.70 | 0.285 | 4.61 | 0.15 – 44.21 | 0.385 |
| Childhood Abuse at 8 years | 0.93 | 0.56 – 1.54 | 0.770 | 0.94 | 0.56 – 1.57 | 0.813 |
| Friendship at 8 years** | **1.12** | **1.06-1.18** | **<0.001** | **1.12** | **1.06 – 1.18** | **<0.001** |
| Self-Esteem at 8 years | 1.06 | 0.99 – 1.13 | 0.084 | 1.05 | 0.99 – 1.12 | 0.121 |
| Locus of Control at 8 years | 1.01 | 0.99 – 1.02 | 0.397 | 1.00 | 0.99 – 1.01 | 0.512 |
| WISC Total IQ at 8 years | **0.99** | **0.98 – 0.99** | **0.005** | **0.99** | **0.98 – 0.99** | **0.008** |
| Art Engagement at 9 years | 0.93 | 0.85 – 1.01 | 0.090 | 0.93 | 0.86 – 1.02 | 0.134 |
| C-Reactive Protein at 9 years | 0.99 | 0.90 – 1.07 | 0.740 | 0.98 | 0.90 – 1.06 | 0.587 |
| Interleukin-6 at 9 years | 0.91 | 0.60 – 1.38 | 0.657 | 0.91 | 0.60 – 1.40 | 0.677 |
| Total Sleep During Night at 9 years | 0.91 | 0.73 – 1.14 | 0.405 | 0.87 | 0.69 – 1.08 | 0.212 |
| Bedtime at 9 years | 1.09 | 0.87 – 1.37 | 0.456 | 1.10 | 0.87 – 1.38 | 0.420 |
| Religion at 9 years | 0.99 | 0.71 – 1.37 | 0.945 | 0.92 | 0.65 – 1.28 | 0.609 |
| Attention Skysearch at 10 years | 0.96 | 0.90 – 1.04 | 0.345 | 0.96 | 0.89 – 1.04 | 0.337 |
| Attention Dualtask at 10 years | 0.96 | 0.92 – 1.07 | 0.088 | 0.97 | 0.93 – 1.01 | 0.177 |
| Attention Opposite Worlds Task at 10 years | **2.66** | **1.33 – 5.30** | **0.005** | **2.17** | **1.07 – 4.40** | **0.033** |
| Loneliness at 10 years | **1.51** | **1.14 – 2.01** | **0.004** | **1.51** | **1.13 – 2.01** | **0.005** |
| School Connectedness at 11 years** | **1.14** | **1.10 – 1.18** | **<0.001** | **1.15** | **1.11 – 1.19** | **<0.001** |
| School Enjoyment at 11 years** | **1.14** | **1.06 – 1.24** | **<0.001** | **1.19** | **1.10 – 1.29** | **<0.001** |
| Participation outdoor activities at 11 years | **0.61** | **0.41 – 0.92** | **0.019** | **0.62** | **0.41 – 0.93** | **0.021** |

OR = odds ratio

*Adjusted model controlled for sex, ethnicity, SES, temperament at 2 years and preterm, and maternal postnatal depression at 8 months.

**These variables were invertedly coded, with higher scores indicating worse outcomes, and lower scores better outcomes.

**Table S7.** Associations between combined factors and persistent high levels of depressive symptoms, without loneliness item.

| **Persistent high levels of depressive symptoms** | | | | | | |
| --- | --- | --- | --- | --- | --- | --- |
|  | Unadjusted model | | | Adjusted model* | | |
|  | OR | 95% CI | p | OR | 95% CI | p |
| Male Parenting Score at 81 months** | 1.02 | 0.99 – 1.05 | 0.147 | 1.02 | 0.99 – 1.05 | 0.198 |
| Friendship at 8 years** | 0.95 | 0.84 – 1.07 | 0.403 | 0.94 | 0.83 – 1.06 | 0.303 |
| WISC – Total IQ at 8 years | 1.00 | 0.98 – 1.02 | 0.995 | 1.00 | 0.98 – 1.02 | 0.914 |
| Total Sleep During Night at 9 years | 1.55 | 0.58 – 4.18 | 0.384 | 1.62 | 0.60 – 4.39 | 0.344 |
| Bedtime at 9 years | 1.53 | 0.52 – 4.45 | 0.436 | 1.48 | 0.50 – 4.37 | 0.479 |
| **Loneliness at 10 years** | **2.65** | **1.53 –4.57** | **<0.001** | **2.48** | **1.40 – 4.38** | **0.002** |
| **School Connectedness at 11 years**** | **1.17** | **1.07 – 1.28** | **<0.001** | **1.18** | **1.08 – 1.30** | **<0.001** |
| School Enjoyment at 11 years** | 0.85 | 0.70 – 1.05 | 0.147 | 0.88 | 0.71 – 1.09 | 0.231 |

OR = odds ratio

*Adjusted model controlled for sex, ethnicity, SES, temperament at 2 years, preterm and maternal postnatal depression at 8 months.

**These variables were invertedly coded, which higher scores indicating worse outcomes, and lower scores better outcomes. Note1: The selection of these risk factors was done based on lived experience involvement.

**Table S8.** Associations between statistically significant factors and persistent high levels of depressive symptoms from 12.5 months to 22 years, without loneliness item.

| **Persistent high levels of depressive symptoms** | | | | | | |
| --- | --- | --- | --- | --- | --- | --- |
|  | Unadjusted model | | |  | Adjusted model | |
|  | OR | 95% CI | p | OR | 95% CI | p |
| Male Parenting Score at 81 months | 0.99 | 0.97 – 1.02 | 0.688 | 0.99 | 0.97 – 1.02 | 0.820 |
| Friendship at 8 years | 0.96 | 0.85 – 1.08 | 0.472 | 0.94 | 0.83 – 1.07 | 0.363 |
| WISC – Total IQ at 8 years | 1.00 | 0.98 – 1.02 | 0.769 | 1.00 | 0.98 – 1.02 | 0.723 |
| Attention Opposite Worlds Task at 10 years | 0.79 | 0.59 – 1.06 | 0.115 | 0.74 | 0.54 – 1.01 | 0.154 |
| **Loneliness at 10 years** | **2.42** | **1.47 – 3.99** | **<0.001** | **2.29** | **1.36 – 3.85** | **0.002** |
| **School Connectedness at 11 years** | **1.15** | **1.05 – 1.26** | **0.002** | **1.16** | **1.06 – 1.28** | **0.001** |
| School Enjoyment at 11 years | 0.86 | 0.70 – 1.06 | 0.170 | 0.87 | 0.70 – 1.08 | 0.199 |
| Participation outdoor activities at 11 years | 0.92 | 0.46 – 1.82 | 0.812 | 0.88 | 0.44 – 1.77 | 0.717 |

**Note1: Here we included all the risk factors that appeared statistically significant in the regression analyses conducted with each risk factor in separate model (i.e., Table S5).

# Further details of the ALSPAC cohort

The initial number of pregnancies enrolled was 14,541 (for these at least one questionnaire was returned, or a “Children in Focus” clinic had been attended by 19/07/99). Of these initial pregnancies, there was a total of 14676 foetuses, resulting in 14062 live births and 13988 children who were alive at 1 year of age. When the oldest children were approximately 7 years of age, an attempt was made to bolster the initial sample with eligible cases who had failed to join the study originally. As a result, in our study, as some variables were collected from the age of seven onwards there were data available for more than the 14541 pregnancies mentioned above. The number of new pregnancies not in the initial sample (known as Phase I enrolment) that are currently represented in the released data and reflecting enrolment status at the age of 24 is 906, resulting in an additional 913 children being enrolled (456, 262 and 195 recruited during Phases II, III and IV respectively). The total sample size for analyses using any data collected after the age of seven is therefore 15,447 pregnancies, resulting in 15,658 foetuses. Of these 14,901 children were alive at 1 year of age. Informed consent for the use of data collected via questionnaires and clinics was obtained from participants following the recommendations of the ALSPAC Ethics and Law Committee at the time. Ethical approval was obtained from the ALSPAC Law and Ethics committee and the local research ethics committees.

Study data were collected and managed using REDCap electronic data capture tools hosted at the University of Bristol (Harris *et al.*, 2009). REDCap (Research Electronic Data Capture) is a secure, web-based software platform designed to support data capture for research studies.

Please note that the study website contains details of all the data that is available through a fully searchable data dictionary and variable search tool" and reference the following webpage: http://www.bristol.ac.uk/alspac/researchers/our-data/.

**References**

Harris, P. A. *et al.* (2009) ‘Research electronic data capture (REDCap)-A metadata-driven methodology and workflow process for providing translational research informatics support’, *Journal of Biomedical Informatics*, 42(2), pp. 377–381. doi: 10.1016/j.jbi.2008.08.010.
